# Surgical treatment of intracapsular temporomandibular disorders

**DOI:** 10.2340/aos.v83.40633

**Published:** 2024-05-07

**Authors:** Bailing Chen, Xinhua Qu

**Affiliations:** a Muping District Traditional Chinese Medicine Hospital, Yantai, China; b GYN Department, Yantai Affiliated Hospital of Binzhou Medical University, Yantai, China

**Keywords:** Pathology, temporomandibular joint, surgical methods, conservative methods, functional disorders

## Abstract

**Objective:**

Temporomandibular joint (TMJ) pathologies are prevalent, affecting approximately 40% of the worldwide population, with nearly 80% involving intracapsular disorders. Despite this, standardized treatment protocols are lacking. This study aimed to compare the efficacy of conservative and surgical approaches in managing intracapsular TMJ disorders.

**Methods:**

Eighty-six patients diagnosed with intracapsular TMJ disorders were included in the study, with 40 males and 46 females, averaging 52.4 ± 4.7 years. Patients were recruited from polyclinics in Beijing, China (*n* = 36), and Kyiv, Ukraine (*n* = 50). A comprehensive examination protocol was conducted, including assessment of patient complaints, medical history, jaw mobility measurements, TMJ palpation, and magnetic resonance imaging (MRI) screening.

**Results:**

The main outcomes of our study revealed significant improvements in patients undergoing surgical intervention for intracapsular TMJ disorders, particularly in cases of disc displacement. Conservative mouth guard/occlusal splint treatment showed limited effectiveness, primarily improving joint effusion and disc displacement. Surgical intervention led to notable enhancements in various TMJ parameters, with significant improvements observed in joint function and pain reduction. Based on these findings, orthodontic rehabilitation was recommended to ensure long-term efficacy, focusing on optimizing occlusion and restoring TMJ function. These results highlight the importance of tailored treatment approaches for managing intracapsular TMJ disorders, emphasizing the role of surgical intervention coupled with comprehensive rehabilitation strategies.

**Conclusions:**

Future research should consider demographic factors and explore innovative examination methods, such as optical systems, to enhance understanding and management of intracapsular TMJ disorders.

## Introduction

Approximately 40% of the temporomandibular joint (TMJ) worldwide patient population suffers from pathologies, with nearly 80% of them being intracapsular disorders [1]. These pathologies affect both the bone and soft tissues within the TMJ capsule. Doctors typically resort to conservative procedures for diagnosing such abnormalities. However, regarding surgical interventions, information about their effectiveness in comprehensive treatment is scarce and conflicting. Some researchers suggest that physiotherapy combined with occlusal stabilization treatment is the most effective approach [2], while others argue that conservative (non-surgical) methods have limitations [3]. For instance, patients undergoing conservative mouth guard/occlusal splint treatment may experience relapses. To date, no consensus exists regarding the effectiveness of conservative versus surgical techniques, and there is a lack of a unified rehabilitation protocol for intracapsular disorder patients. This poses a significant concern, as TMJ pathologies are prevalent worldwide and often challenging to diagnose at an early stage, particularly concerning joint damage. Additionally, there is a need to compare the various treatments available for TMJ disorders in terms of their effectiveness [4]. The results of such a comparative analysis could contribute to the development of a standardized treatment protocol.

Over the past two decades, there have been notable advancements in maxillofacial surgery and dentistry, particularly in the conservative and surgical treatment of TMJ pathologies, including minimally invasive procedures. Surgical intervention for TMJ disorders is typically recommended in cases involving rheumatic diseases, necessitating an interdisciplinary approach. It is crucial, in accordance with studies, that surgical procedures should be reserved for patients with low disease activity or those in remission [5]. TMJ pathologies frequently coincide with rheumatic conditions, notably rheumatoid arthritis (RA) [1]. Incidence rates in RA patients vary widely, ranging from 5% to 88% [2], with disease duration correlating with TMJ severity [3]. Acute asymmetric TMJ arthritis affects about 20% of RA patients at disease onset, leading to pain and reduced jaw function [4]. In psoriatic arthritis, TMJ involvement occurs in over 50% of cases [5], with initial pathology seen in approximately 27% of patients [6]. While TMJ issues can manifest in ankylosing spondylitis, data are limited [7] although connective tissue dysplasia is diagnosed in 5% to 35% of these patients [8]. Systemic lupus erythematosus patients often report TMJ pain, while systemic scleroderma sufferers may experience limited mouth opening [9].

Wilkes stages IV and V, identified through CT and magnetic resonance imaging (MRI), signify advanced degeneration in TMJ bone and cartilage, leading to joint deformities and potential disc displacement [10]. Surgical risks are debated [11], with minimally invasive methods gaining traction [12]. Conservative mouth guard/occlusal splint treatments’ efficacy varies, with complications or failures ranging from 8% to 60% [13], often resulting in relapse.

A substantial portion of TMJ disorders relate to the dentoalveolar system, encompassing various dysfunctions and diseases [14]. TMJ pain shares similarities with cervical neuralgia and other conditions [15]. Diagnostic methods include X-ray radiography, arthroscopy, CT, and 3D modeling, offering varying degrees of efficacy [16]. Orthopantomography is commonly used despite limitations in image distortion and accuracy [17].

### Problem statement

Comparative studies evaluating the effectiveness of surgical and conservative (non-surgical) interventions in treating TMJ pathologies remain underrepresented in modern medicine. A standardized patient examination protocol that simplifies both the diagnostic process and treatment strategy is conspicuously absent. Furthermore, TMJ pathologies are often diagnosed in advanced stages, necessitating orthodontists to consider the extent of disease involvement. This issue serves as the foundation for the current research. The present study provides a comparative analysis of conservative and surgical techniques for treating TMJ pathology. Our hypothesis posits that, despite its drawbacks, surgical intervention is more effective than conservative mouth guard/occlusal splint treatment. This study aims to compare the effectiveness of conservative and surgical treatment approaches for managing intracapsular TMJ disorder. The study objectives encompass the following: (1) conducting primary patient evaluations, including assessing jaw opening, range of jaw motion, and TMJ pain; (2) performing functional assessments of the TMJ; (3) implementing a conservative mouth guard/occlusal splint treatment approach and evaluating patient outcomes at 3 and 6 months; and (4) conducting appropriate surgical interventions whenever feasible.

## Methods

### Participants

The study encompassed 86 patients diagnosed with intracapsular TMJ disorders, comprising 40 male and 46 female patients, with a mean age of 52.4 ± 4.7 years. The study was conducted in 2022 across two polyclinics: one in Beijing, China (PRC; 36 patients), and the other in Kyiv, Ukraine (50 patients) (all in local Departments of Maxillofacial Surgery). The Chinese patients had a mean age of 51.5 ± 4.4 years, while the Ukrainian patients had a mean age of 52.6 ± 4.9 years. All participants underwent a standardized examination protocol, which included a comprehensive assessment of patient complaints, medical history, specific measurements such as the degree of jaw opening, range of jaw movement (laterotrusion and protrusion), palpation of the TMJ and maxillofacial muscles, and MRI screening.

Thirteen patients who declined to participate were excluded from the study, in addition to the 86 who consented to participate.

Participants were identified during regular dental visits at the selected outpatient clinics. Patients meeting the inclusion criteria were invited to participate in the study. The recruitment process did not involve random sampling; instead, it relied on the identification of individuals with symptoms of intracapsular TMJ disorders during dental appointments.

#### Selection criteria

The criteria for participants in this prospective cohort study included individuals who presented complaints of pain in the TMJ region and restricted jaw mobility due to intracapsular TMJ disorders. Exclusion criteria encompassed patients with a history of TMJ surgeries, systemic autoimmune diseases affecting the TMJ, and contraindications to the selected treatment modalities. Participants were recruited from two outpatient clinics: one in Beijing, the People’s Republic of China (PRC), and one in Kyiv, Ukraine.

### Instruments and procedures

The Helkimo Clinical Dysfunction Index (HCDI) is a clinical scale employed for the evaluation of functional disturbances within the TMJ domain. The Helkimo scale assesses various aspects of TMJ dysfunction, encompassing pain, range of motion limitations, clicking, and other related symptoms. Evaluation is conducted by assigning scores to the presence and severity of each of these symptoms, which are subsequently aggregated to yield an overall assessment of TMJ dysfunction.

Range of Protrusion Movement (RPM): This scale quantifies the extent of forward movement of the lower jaw. It represents a critical parameter in the assessment of TMJ function. Limitations in the range of forward movement can arise due to various factors, including joint dysfunction or the presence of painful symptoms.

Temporomandibular Joint Internal Derangement Scale (TIDS): This scale is utilized for the assessment of internal alterations within the joint. It may encompass the evaluation of parameters such as disc position, the presence of inflammation, and other anatomical and functional aspects of the joint. The assessment using TIDS can aid in determining the degree of internal changes in the TMJ and their impact on overall joint function.

Visual Analog Scale (VAS): The VAS is employed for the measurement of pain intensity. Patients are asked to place a mark on a line that represents a spectrum ranging from the absence of pain to maximum pain intensity. This is a widely utilized and validated method for assessing pain sensations.

The Wilkes Classification System: This assessment system is utilized for the categorization of various degrees of degeneration and structural alterations in the TMJ, including the articular disc and joint surfaces. It aids in determining the severity of changes within the joint and can be valuable in assessing the effectiveness of treatment.

Therefore, various scales and assessment methods were employed in the study to comprehensively and objectively investigate the condition and function of the TMJ in patients.

All patients were assessed to determine the severity of dysfunction using the Helkimo Craniomandibular Disorder Index (HCDI). The HCDI categorizes patients into different groups based on their scores: clinically symptom free (0 points), mild dysfunction (1 to 4 points), moderate dysfunction (5 to 9 points), and severe dysfunction (10 to 25 points). Pain localized in the TMJ area was assessed using the Rocabado pain map (RPM), while pain intensity in the TMJ area was measured using the VAS. Functional TMJ assessments included the measurement of maximum jaw opening distance, lateral jaw movement (laterotrusion) on both sides, and protrusive jaw movement (protrusion). The degree of jaw opening was determined by measuring the distance between the incisal edges of the maxillary and mandibular teeth during rotation/translation movement.

Patients with functional limitations of the mandible underwent MRI and cone-beam computed tomography (CBCT) screening both at baseline and at the 6-month follow-up. MRI scans were performed in both open- and closed-mouth positions. The MRIs were carefully examined for various TIDS characteristics, including joint effusion, disc displacement, disc nonrecapture, disc degenerative changes, abnormal condyle translation, and condyle arthritis. TIDS is a novel MRI imaging assessment score used to evaluate clinical TMJ pathology.

- The range of anterior displacement of the mandible (RPM) was 35–40 mm, based on commonly accepted standards for assessing TMJ function. The VAS was applied with ratings ranging from 0 to 10, where 0 indicates the absence of pain, and 10 denotes the maximum intensity of pain sensations.
- In measuring the maximum jaw opening, vertical overbite was taken into account to ensure accurate assessment results of the functional activity of the TMJ.

We characterized pain as the overall intensity over the last 30 days, intensity during movements and palpation, and also assessed the current pain intensity at the time of examination. This allowed us to obtain a comprehensive understanding of the pain symptoms in patients with intracapsular TMJ disorders.

### Surgical interventions

Surgical interventions, specifically open joint disc repositioning, were performed on 28 patients. The indications for surgery included joint disc displacement with or without deformity or condyle head displacement that did not respond to conservative mouth guard/occlusal splint treatments, severe TMJ pain leading to restricted mouth opening, jaw locking, mandibular movement asymmetry, abnormalities in anatomical structures surrounding the TMJ detected by CT scans, and joint space narrowing observed on MRI scans. Clinical and radiological abnormalities were classified according to the Wilkes criteria.

The Method of Open Repositioning of the Articular Disc of the TMJ (Open Diskectomy and Repositioning) [18, 19].

Open repositioning of the articular disc of the TMJ is a surgical procedure aimed at restoring the displaced or dislocated articular disc within the TMJ, commonly used to treat conditions like articular disc dislocation and arthrosis.

The procedure involves:

The patient receives local or general anesthesia for comfort and pain reduction.

A small incision is made in front of the ear to access the TMJ.

The TMJ is carefully examined to assess the condition of the articular disc and other structures.

If displaced, the surgeon gently repositions the articular disc, potentially removing compressed or damaged tissues.

After all necessary manipulations, the incision is closed with sutures or adhesive materials.

The patient receives instructions for postoperative care, including diet, pain management, and rehabilitation.

This surgical intervention aims to restore the normal anatomy and function of the TMJ, which can significantly alleviate symptoms of TMJ pathologies such as pain, jaw mobility limitations, and joint sounds.

The indications for surgery included joint disc displacement with or without deformity or condyle head displacement that did not respond to conservative mouth guard/occlusal splint treatments, severe TMJ pain leading to restricted mouth opening, jaw locking, mandibular movement asymmetry, abnormalities in anatomical structures surrounding the TMJ detected by CT scans, and joint space narrowing observed on MRI scans. Clinical and radiological abnormalities were classified according to the Wilkes criteria.

### Conservative mouth guard/occlusal splint treatment

**Figure 1:**
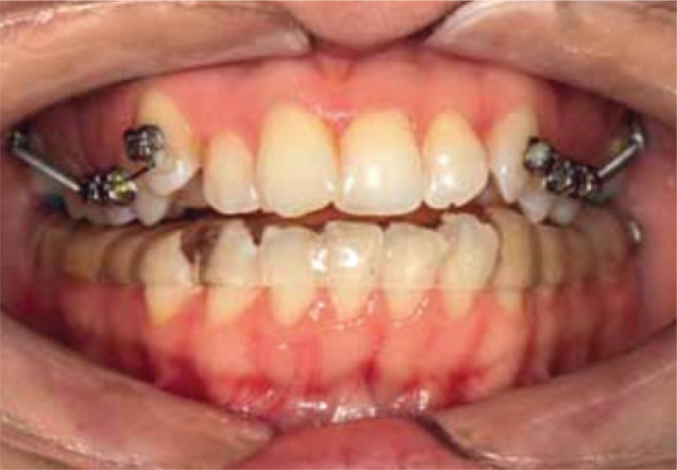
A dental mouth guard used for a conservative mouth guard/occlusal splint treatment.

All patients initially underwent conservative therapy, which involved the use of a lower jaw dental mouth guard for a duration of 3 to 4 months (Figure 1).

Patients were re-evaluated after the conservative mouth guard/occlusal splint treatment period. If a positive response to conservative mouth guard/occlusal splint treatment was not observed, patients meeting specific criteria were offered surgical intervention as a therapeutic option.

**Figure 2:**
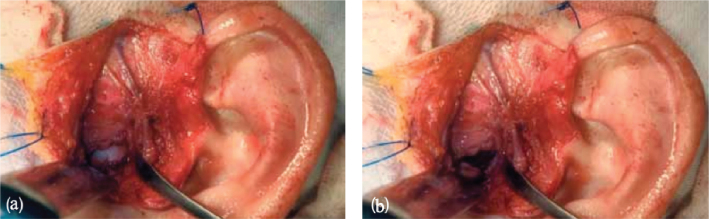
TMJ disc repositioning through an open incision in closed-jaw (a) and open-jaw (b) positions.

The surgical procedures aimed to improve the mechanical and functional properties of the TMJ, increase the range of jaw motion, and alleviate pain. For this purpose, patients underwent articular disc repositioning (Figure 2).

However, it is worth noting that according to recommendations, TMJ pain problems should be addressed with a combination of various conservative methods before a reliable evaluation of treatment outcomes. Therefore, it is important to consider incorporating a multidimensional conservative mouth guard/occlusal splint treatment regimen encompassing a range of therapeutic modalities tailored to individual patient needs and symptoms to optimize treatment efficacy. Subsequent re-evaluation postconservative mouth guard/occlusal splint treatment would then be conducted to assess patient response before considering surgical intervention as a therapeutic option.

### Study design

In this study, a prospective cohort design was employed to compare the effectiveness of conservative and surgical treatment methods in the management of intracapsular TMJ disorders. Participants were classified based on the severity of their condition, and treatment procedures were assigned according to this classification.

Criteria used to determine non-response to conservative mouth guard/occlusal splint treatment were based on the clinical assessment of symptoms in patients and their progression during the treatment period. Patients were considered nonresponsive to conservative mouth guard/occlusal splint treatment if they did not exhibit significant improvement in TMJ symptoms, as evidenced by a reduction in pain, improved jaw mobility, and resolution of other issues related to intracapsular TMJ disorders. The study assessed treatment outcomes over 6 months following treatment.

### Statistical analysis

Data analysis was performed in Statistica v. 10 (Statsoft Inc., USA). Results are presented as means and standard errors of the mean. Student’s t-test was employed to compare the outcomes of two treatments. In our study, Student’s t-test was chosen for the analysis of differences between the two groups. This choice was substantiated by the observation that our data exhibited an approximate normal distribution, and preliminary tests for equality of variances also did not reveal statistically significant differences in variability between the groups. Furthermore, our research question directly entailed comparing the means between groups, making Student’s *t*-test an appropriate tool for this analysis. The differences were considered significant at an alpha level of ≤ 0.05. The level of statistical power reached 0.8.

Dependent variables:

Treatment efficacy in cases of intracapsular TMJ disorders.

Changes in HCDI scores.

Changes in the VAS for assessing pain in the TMJ region.

Changes in the structure of the articular disc and other pathological features on MRI scans.

Independent variables:

Type of treatment (conservative or surgical).

Age of the patients.

Gender of the patients.

Location of the study (outpatient clinics in Kyiv, Ukraine, and Beijing, China).

Year of the study (2022).

### Ethical statement

This study respects the international standards of ethics and morality. All patients gave written consent to participate in the study and to use conservative and surgical methods of treatment. Anonymity and confidentiality were guaranteed. In case of ineffectiveness of conservative mouth guard/occlusal splint treatment, additional consultations were carried out to inform patients about a surgical alternative. The research protocol was approved by the Ethics Committee of Yantai Affiliated Hospital of Binzhou Medical University (Protocol No. 449) and by the Ethics Committee of the Luhansk State Medical University (Protocol No. 229-1).

## Results

The patients were evaluated with the Helkimo index, TIDS, VAS, RPM, and Wilkes classification system. Results were interpreted using the scheme presented in Table 1.

**Table 1:** Interpretation of clinical and radiological findings.

Degree of TMJ dysfunction	HCDI, points	TIDS, points	Wilkes, stage	VAS, points	Pain localization
None	0	0–1	0	0–2	Superior anterior and/or Superior Posterior Synovials (pain #2 and #6)
Mild	1–4	1–2	1	3–4	Anterior Inferior Synovial and/or Lateral Collateral Ligament (pain #1 and #3)
Moderate	5–9	3–4	2 and 3	5–6	Temporomandibular Ligament and/or Bilaminar Zone (pain #4 and #7)
Severe	10–25	5–6	4 and 5	7–10	Anterior Inferior and/or Posterior Inferior Synovials and/or Retrodiscal Tissue (pain #1, #5 and #8)

HCDI: Helkimo Clinical Dysfunction Index; VAS: Visual Analog Scale; TMJ: temporomandibular joint; TIDS: Temporomandibular Joint Internal Derangement Scale.

The proposed scheme allows for data correlation across scales and assessments, enabling researchers to estimate the degree of TMJ damage. The analysis of surgical treatment outcomes suggests improvement. The results are presented in Tables 2 and 3.

**Table 2:** The comparison of HCDI, TIDS, and VAS scores and the maximum jaw opening distance in patients before and 6 months after surgical intervention.

Variables	Before surgery	Six months after surgery
HCDI score	16.5 ± 1.6	6.3 ± 1.1*
TIDS score	5.1 ± 2.1	2.2 ± 1.6*
Maximum jaw opening distance, mm	29.4 ± 2.7	43.5 ± 2.6*
VAS score	7.2 ± 2.4	2.3 ± 1.3*

HCDI: Helkimo Clinical Dysfunction Index; VAS: Visual Analog Scale; TIDS: Temporomandibular Joint Internal Derangement Scale.

Note:

* – the differences are significant at *p* < 0.05.

**Table 3:** The distribution of patients according to TIDS scores.

Findings	Baseline (*N* = 86)	After splint therapy (*N* = 86)	Before surgery (*N* = 28)	After surgery (*N* = 28)	Six months after surgery (*N* = 28)
Joint effusion	66.0 ± 2.4	36.0 ± 2.2*	60.0 ± 2.1	10.0 ± 1.3^1,2^	2.0 ± 2.3^3^
Disc displacement	58.0 ± 2.3	44.0 ± 1.1*	55.0 ± 2.0	2.0 ± 0.6^1,2^	None^3^
Disc nonrecapture	28.0 ± 1.4	28.0 ± 1.8**	25.0 ± 1.5	4.0 ± 1.1^1,2^	4.0 ± 1.0
Disc degenerative changes	16.0 ± 2.3	16.0 ± 1.1**	14.0 ± 2.0	12.0 ± 1.6^1^	10.0 ± 1.3
Abnormal condyle translation	24.0 ± 2.6	22.0 ± 1.3**	22.0 ± 2.3	20.0 ± 2.6	None^3^
Condyle arthritis	4.0 ± 1.1	4.0 ± 1.2**	3.0 ± 1.0	None^2^	None^3^

TIDS: Temporomandibular Joint Internal Derangement Scale.

Note:

* – the difference was statistically significant compared to baseline (*р* ≤ 0.05);

** – the difference was statistically insignificant compared to baseline (*р* ≥ 0.05);

1,2 the difference was statistically significant compared to baseline (*р* ≤ 0.05) and conservative mouth guard/occlusal splint treatment (*р* ≤ 0.01), respectively;

3 the difference was statistically significant compared to immediate post-surgical assessment (*р* ≤ 0.01).

Data in Tables 2 show significant differences in all scales and indices, including the maximum jaw opening distance. This finding suggests the effectiveness of the surgical intervention. Table 3 presents the distribution of patients according to the TIDS classification.

According to the data in Table 3, patients who underwent conservative mouth guard/occlusal splint treatment demonstrated significant improvement in only two components of the TIDS score: joint effusion and disc displacement. As for the other TMJ pathologies, conservative mouth guard/occlusal splint treatment failed to improve these symptoms. In contrast, the surgical intervention showed significant improvement in 5 out of 6 components of the TIDS score, with abnormal condyle translation being the only exception. Some parameters improved after 6 months of follow-up; this excludes disc nonrecapture and degenerative changes. Several patients were diagnosed with articular disc displacement (Table 3), including 55 patients treated solely with a mouth guard and 28 patients who underwent surgical intervention.

In this study, oblique-sagittal and oblique-coronal MRI images of patients were analyzed. The location of the medial and distal poles of the articular disc was assessed in relation to the condyle head. Dynamic imaging was performed in a straight sagittal orientation. The following variables were taken into account: the expected trajectory of the condyle, the shape of the condyle head, and its size. Consequently, significant differences in the degree of TMJ dysfunction were established. In particular, the HCDI score decreased by almost 3-fold. The results are presented in Tables 4 and 5.

**Table 4:** The pre-operative MRI findings.

Planes of imaging	Open-jaw positions			Closed-jaw positions		
	3-o’clock position	12-o’clock position	9-o’clock position	3-o’clock position	12-o’clock position	9-o’clock position
Oblique sagittal	None	4	72	None	2	74
Oblique coronal	None	6	70	None	2	74

MRI: magnetic resonance imaging.

Note: The number of patients is given according to the position of the disk in relation to the condyle process.

**Table 5:** The post-operative MRI findings.

Planes of imaging	Open-jaw positions			Closed-jaw positions		
	3-o’clock position	12-o’clock position	9-o’clock position	3-o’clock position	12-o’clock position	9-o’clock position
Oblique sagittal	72	4	None	74	2	None
Oblique coronal	72	4	None	None	74	2

MRI: magnetic resonance imaging.

Changes in the HCDI score suggest a clinically significant improvement (Table 5, *p* ≤ 0.05). This average outcome can be classified as mild TMJ dysfunction (Tables 2).

The maximum jaw opening distance increased by 1.5-fold (*p* ≤ 0.05); this change is considered significant. There were also significant differences in TIDS scores (2.5-fold increase, *p* ≤ 0.05; Tables 2).

The surgical procedures primarily involved the repositioning of the articular disc within the TMJ. This entailed gently relocating the displaced or dislocated disc to its normal anatomical position. In some cases, it also involved the removal of compressed or damaged tissues surrounding the disc. The specific procedures performed varied based on the individual patient’s condition and requirements. However, it is essential to note that the outcome may have been influenced by the type of procedure undertaken although further investigation or data analysis would be needed to establish a definitive correlation.

The main outcome of the surgical intervention was to restore the normal anatomical alignment and functional properties of the TMJ. This encompassed alleviating symptoms such as pain, restricted jaw mobility, and joint dysfunction associated with conditions like articular disc displacement and arthrosis. The ultimate goal was to improve the overall quality of life and functional capacity of the patients affected by TMJ pathologies.

## Discussion

Treating TMJ osteoarthritis represents a significant challenge in contemporary orthopedic dentistry [20, 21]. Our results confirm a substantial number of patients presenting with symptoms of osteoarthritis. There are two primary approaches to treatment: occlusal correction to restore the position of the mandible and eliminate dental deformities [22], as well as stimulation of bone and cartilage metabolism in the joint through local pharmacological treatment and physiotherapy [23, 24]. Our study aligns with previous research indicating the limited effectiveness of removable protective devices compared to surgical interventions [25]. Removable protective devices demonstrated lower efficacy, improving only two parameters of the TIDS scale. In contrast, surgical disc restoration improved five parameters of the TIDS scale [26, 27], maintaining progress for up to 6 months postoperation.

In our study, an open articulatory disc restoration procedure was employed, which proved to be effective, although we also noted potential complications such as scarring or facial nerve damage [28]. CBCT and MRI were fundamental components for diagnosing TMJ pathologies [24, 29]. While various dental assessment methods exist, such as electromyography and rheoarthrography, they were not utilized in our study. Successful TMJ treatment requires a comprehensive evaluation, considering both clinical and paraclinical methods. While our study predominantly utilized standard assessments such as MRI and CT, it underscores the effectiveness of surgical intervention compared to conservative treatment in the form of removable protective devices for managing TMJ pathologies.

Potential sources of bias may include selective exclusion of participants or sampling restrictions in certain groups. These factors can introduce distortions in result interpretation. For future research, it is important to consider stricter selection criteria and broaden geographical coverage to ensure representativeness. When assessing the strengths and weaknesses of the study, it is crucial to highlight the effectiveness of the methods employed, such as open articulatory disc restoration. Nevertheless, it is important to acknowledge limitations such as a small sample size, which may limit the generalizability of the results.

Future research could focus on examining other potential factors, such as the severity of symptoms or tissue degeneration, to gain a deeper understanding of their impact on treatment outcomes. Additionally, further exploration of approaches that combine conservative treatment in the form of removable protective devices with surgical interventions could be warranted to determine which combinations of methods are most effective in specific cases. These propositions may help identify potential avenues for further research and improvement in the treatment of patients with intracapsular TMJ disorders.

## Conclusions

The research findings underscore the high efficacy of open repositioning of the articular disc in the treatment of intracapsular TMJ pathologies. Surgical intervention proved to be more successful (96%) compared to capsulotomy (24%), resulting in an improvement of the articular disc condition and a reduction in degeneration. Orthodontic rehabilitation is recommended to maintain progress and normalize occlusion.

## Data Availability

Data will be available on request.
